# Evaluation of Needles in Endoscopic Ultrasound-Guided Tissue Acquisition of Pancreatic Cancer for Genetic Yield and Quality

**DOI:** 10.7759/cureus.68431

**Published:** 2024-09-02

**Authors:** Jonathan Tiong, Phi Nguyen, Mithra Sritharan, Joanne Lundy, Henry Shen, Beena Kumar, Michael Swan, Brendan Jenkins, Daniel Croagh

**Affiliations:** 1 Department of Surgery, Monash Health, Melbourne, AUS; 2 Centre for Innate Immunity and Infectious Diseases, Hudson Institute of Medical Research, Melbourne, AUS; 3 Department of Anatomical Pathology, Monash Health, Melbourne, AUS; 4 Department of Gastroenterology, Monash Health, Melbourne, AUS

**Keywords:** needle size, needle type, genetic yield, pancreatic cancer, endoscopic ultrasound guided needle biopsy

## Abstract

Background: Endoscopic ultrasound-guided fine needle biopsy (FNB) is the gold standard in tissue acquisition of pancreatic ductal adenocarcinoma (PDAC). There is a paucity of evidence of the impact of needle type or size on the genetic yield and quality.

Methods: Patients 18 years and older with PDAC who underwent FNB were retrospectively identified from a single database from 2016 to 2021. Genetic quantity is measured in micrograms (µg) and quality defined by RNA or DNA integrity number (RIN and DIN). FNB needles examined were Acquire 22 gauge (Boston Scientific, Marlborough, MA, USA) and ProCore 22 and 20 gauges (Cook Medical, Bloomington, IN, USA).

Results: Two hundred seventy-seven patients were identified. ProCore 20G needle procured higher RNA quantity (4125.8µg, IQR: 2003.8, 5954.8, p = 0.012) compared to ProCore 22G (2050µg IQR: 966.4, 3181.6) and Acquire 22G (2310.6µg, IQR: 1439.3, 4312). Median DNA quantity was 3340.5µg (Acquire 22G), 2610.4µg (ProCore 22G) and 3499.7µg (ProCore 20G) (p = 0.763). Median DIN was 7.3 (Acquire 22G and ProCore 22G) and 7.4 (ProCore 20G) (p = 0.449). Median RIN was 3.0 (Acquire 22G and ProCore 22G) and 2.7 (ProCore 20G) (p = 0.886).

Conclusion: ProCore 20G was associated with higher quantity of RNA. There were no differences in the quality acquired by different needles.

## Introduction

Pancreatic cancer is among the most aggressive malignancies, associated with a poor 5% to 15% five-year survival rate. Pancreatic ductal adenocarcinoma (PDAC) is the most common variant and is typically asymptomatic in its early stages [1]. Although surgery is the only chance of cure, due to its late presentation, only 20% of patients with PDAC have surgically resectable disease [2]. Additionally, while chemotherapy agents have managed to improve the survival of patients with early-stage pancreatic cancer, this improvement in mortality and morbidity is not reflected in later-stage disease [3]. Therefore, recent trends are seeing a shift to incorporate immunotherapy-based strategies to target stepwise events in tumour initiation and progression, otherwise known as precision medicine. Targeted approaches in cancer management have been met with great success in various cancer types including BRAF in breast cancer or EGFR and KRAS in lung cancer [4]. The key component of precision medicine is the extraction of high-quality genetic material in adequate quantities for genomic profiling analysis. One of the main barriers in this area of research, however, is the reliance on the tissue samples that can only be extracted ex vivo (post-operatively or from the resection specimen) from the 20% of surgically resected specimens to isolate genetic material. Unfortunately, this leaves the remaining majority of patients with non-resectable PDAC excluded from genetic analysis [5-7]. For that reason, endoscopic ultrasound-guided tissue acquisition (EUS-TA) plays a pivotal role in providing DNA and RNA for advancing precision medicine, and will likely open up therapeutic options for otherwise unresectable cancers.

Prior to endoscopic ultrasound, endoscopic retrograde cholangiopancreatography (ERCP), percutaneous biopsies and surgical biopsies were used for tissue sampling, though with limitations. EUS-TA has become the gold standard in diagnosing PDAC, and its usage and availability have increased dramatically over the last two decades [8-10]. Studies demonstrate reliable sensitivity and excellent specificity of 85% and 95% respectively [11,12]. Complication rates are also low, at 1% [13]. It involves an endoscopic ultrasound guiding a needle probe through the duodenum to gain direct access to the pancreas, allowing targeted biopsy or aspirate of the suspected lesion.

Indeed, multiple trials cite the difficulty in isolating sufficient amounts of high-quality genomic material for molecular profiling [14-16]. There are a number of factors involved, including needle size and shape, acquisition method (aspirate or biopsy), difference in endoscopist and technical skill. Multiple studies demonstrate the utility of using large needle gauge and biopsy method to acquire more genetic material [17,18]. However, in these earlier studies, only DNA was analysed. While DNA and RNA are procured indiscriminately, RNA is chemically labile and hence more susceptible to degradation, making its acquisition and study more difficult [19]. Despite this, most clinical trials rely on molecular profiling at the DNA level without integrating RNA information. RNA is particularly important for two reasons. First, they are complementary for identifying genomic alterations at the DNA level [20,21]. Second, unlike DNA, RNA execute cellular behaviour, and thus it would be more sensible to target the aberrant source [22]. Ultimately, this principle approach in transcriptomics would allow us to have actionable targets to employ direct intervention in patients [23-25]. To our knowledge, only one study has evaluated factors for higher RNA quality and quantity for molecular profiling. Although their prospective observational investigation yielded no statistically significant difference in needle gauge or approach, they had a small sample size of 37 patients, and results were promising [26]. 

## Materials and methods

Our aim is to compare DNA and RNA quality and quantity to needle types used in EUS-TA. The primary outcome variable is the quality and quantity of genetic material.

Study design

**Figure 1:**
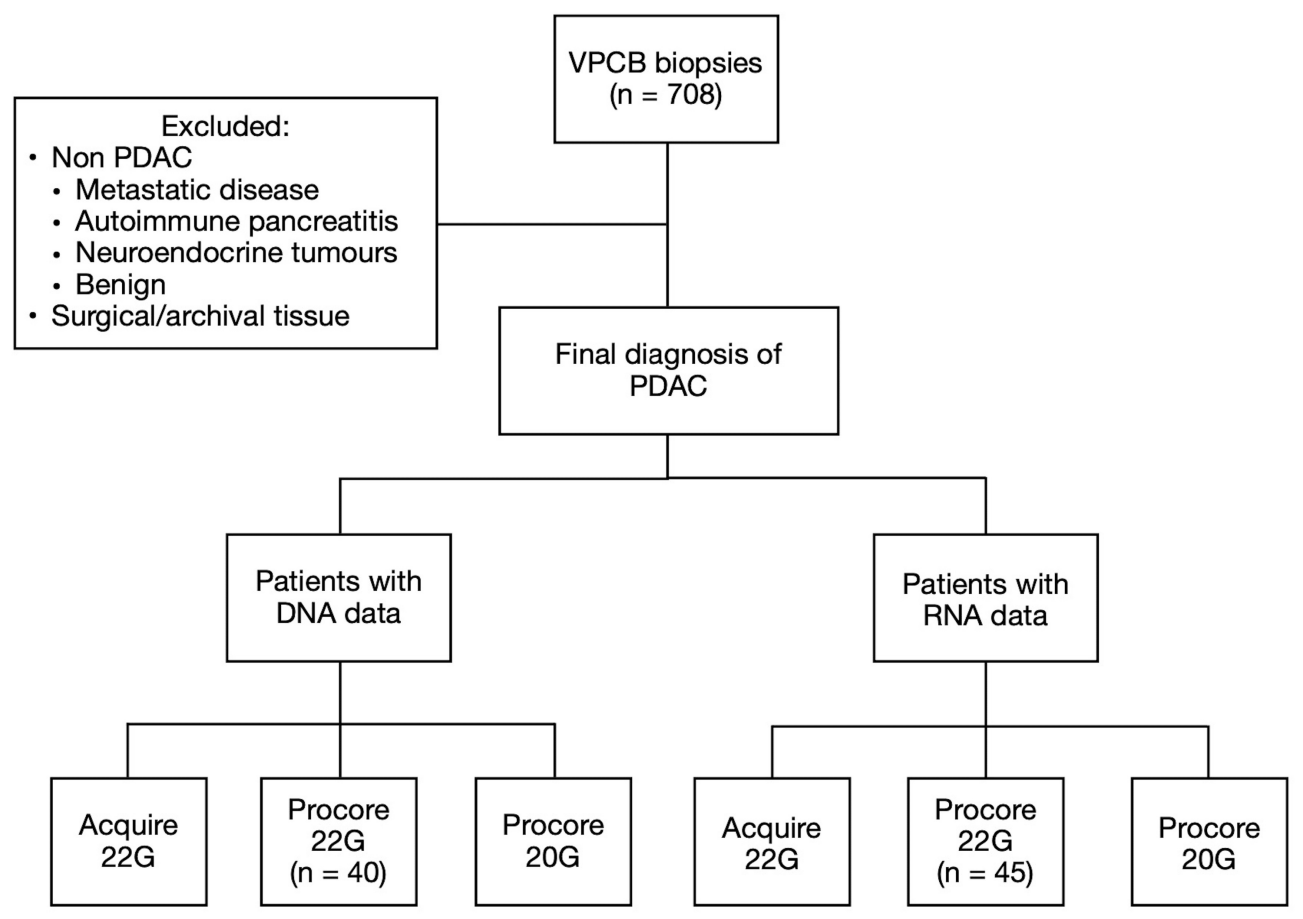
Data collection VPCB=Victorian Pancreatic Cancer Bank; PDAC=Pancreatic ductal adenocarcinoma; DNA=Deoxyribonucleic acid; RNA=Ribonucleic acid

A retrospective case-control study was performed at a single centre in Melbourne, Australia. Patients 18 years and older who underwent EUS-TA and a formal diagnosis of PDAC were included from January 2016 to January 2021. Results of core needle samples were obtained from the Victorian Pancreatic Cancer Biobank. Three main needles were identified (22G Acquire (Boston Scientific, Marlborough, MA, USA), 22G ProCore and 20G ProCore (Cook Medical, Bloomington, IN, USA)) and compared for quality and quantity of DNA and RNA acquired (Figure 1). 

Data collection

Pertinent records examined were: demographics, needle type (20G ProCore, 22G ProCore, 22G Acquire), number of passes, genomic yield and quality, National Comprehensive Cancer Network (NCCN) stage at diagnosis, and site of tumour. Yield is defined by micrograms (μg) and acquired by multiplying the eluted volume (mL) with the NanoDrop (Thermo Fisher, Waltham, MA, USA) concentration (µg/mL), and quality defined by DNA and RNA integrity numbers (DIN and RIN). We excluded patients who did not have a final diagnosis of PDAC (benign, metastatic disease, autoimmune, pancreatitis). In cases where cytology was negative or non-diagnostic, the clinical diagnosis of PDAC was based on clinician consensus at a multidisciplinary meeting. These patients were considered to have sufficient clinical and radiological features to warrant diagnosis and subsequent treatment for PDAC.

Statistical analysis

Continuous variables were reported as mean (standard deviation) or median (range) and categorical variables by proportion. Statistical analysis between three groups utilised Kruskal Wallis test for continuous variables and Fisher’s exact or chi-squared for categorical variables. P values <0.05 were considered significant.

Genomic analysis

Genetic tissues were biopsied from a single pass for research and snap frozen. DNA and RNA were then simultaneously extracted following the protocol according to Qiagen AllPrep DNA/RNA Universal Kit (Hilden, Germany). Quantity was assessed by NanoDrop spectrophotometer, and quality was assessed using TapeStation systems (Agilent, Santa Clara, CA, USA). NanoDrop spectrophotometer measures in concentration values (μg/μL). This was multiplied by the eluted volume specific to each sample. DNA samples from 2016 to July 2017 were eluted in 80μL, July 2017 to December 2019 in 50μL and January 2020 to 2021 in 50μl. RNA samples from 2016 to July 2017 were eluted in 50μL, July 2017 to December 2019 in 40μL and January 2020 to 2021 in 35μl.

The decrease in elution volume is due to the decision to increase DNA and RNA concentration in the eluted solution by using a smaller volume. A higher concentration is advantageous for downstream applications, such as sequencing or quantitative polymerase chain reaction (PCR), which often require more concentrated sample. This was controlled for analysis, therefore does not affect genomic results.

## Results

Demographic and cytology findings for RNA

One hundred forty-five patients with a final diagnosis of PDAC were identified across three needle groups with RNA data (Acquire 22G (n = 43), ProCore 22G (n = 45) and ProCore 20G (n = 57)) (Table 1). Median age between the groups ranged between 68 to 72 (p = 0.438). Acquire 22G had fewer females and ProCore 22G had fewer males (p = 0.021). 

**Table 1:** Demographic and cytology findings for patients with RNA data RNA=Ribonucleic acid; M=male; F=female

RNA (N=145)	Acquire 22G (n=43)	ProCore 22G (n=45)	ProCore 20G (n=57)	p-value
Median Age	68	72	71	0.438
Sex				
M, n (%)	28 (65)	16 (36)	28 (49)	0.021
F, n (%)	15 (35)	29 (64)	29 (51)	
Positive Cytology for PDAC. n(%)	37 (86)	37 (82)	44 (77)	0.522
Other Cytology Findings				
Suspicious, n(%)	1 (2)	2 (4)	2 (4)	0.873
Atypical cells, n(%)	2 (5)	3 (7)	7 (12)	
Non-diagnostic, n(%)	3 (7)	3 (7)	4 (7)	

In total, 118 patients had positive cytology findings for PDAC, five with suspicious or highly suspicious features for PDAC, 12 with atypical cells without definite features of malignancy, and 10 with non-diagnostic cytology. Therefore, the sensitivity of EUS-guided biopsy in diagnosing solid pancreatic masses in this cohort was 81.4%.

The distribution of positive cytology PDAC cases were similar among Acquire 22G (n = 37), ProCore 22G (n = 37) and ProCore 20G (n = 44) (p = 0.522). Non-PDAC cytology findings were similar between groups (p = 0.873). 

RNA tumour characteristics

Majority of patients had either locally advanced (33.1%) or metastatic disease (43.4%). Over half (55.9%) of PDAC tumours were primarily located in the head. Other sites included neck (8.3%), body (15.2%), tail (6.2%), uncinate process (6.9%), distal pancreas (2.1%), and other (bile duct 0.8%). Onsite cytology was available for a significant higher proportion (91%) of ProCore 22G needle biopsies (p < 0.001) (Table 2). 

**Table 2:** Tumor Characteristics RNA=Ribonucleic acid; NCCN=National Comprehensive Cancer Network

RNA (n = 145)	Acquire 22G (n = 43)	ProCore 22G (n = 45)	ProCore 20G (n = 57)	p-value
NCCN Stage				
Operable, n (%)	2 (5)	6 (13)	3 (5)	0.759
Borderline resectable, n (%)	4 (9)	6 (13)	7 (12)	
Locally advanced, n (%)	15 (35)	14 (31)	19 (33)	
Metastatic, n (%)	19 (44)	18 (18)	26 (46)	
Tumour location				
Head, n (%)	18 (42)	30 (67)	33 (58)	0.426
Neck, n (%)	5 (12)	5 (11)	2 (4)	
Body, n (%)	8 (19)	4 (9)	10 (18)	
Tail, n (%)	3 (7)	2 (4)	4 (7)	
Uncinate, n (%)	5 (12)	2 (4)	3 (5)	
Distal, n (%)	1 (2)	1 (2)	1 (2)	
Other, n (%)	0 (0)	0 (0)	2 (4)	
Presence of onsite Cytology, n (%)	5 (12)	41 (91)	12 (21)	<0.001

RNA quality and quantity and correlation

**Figure 2:**
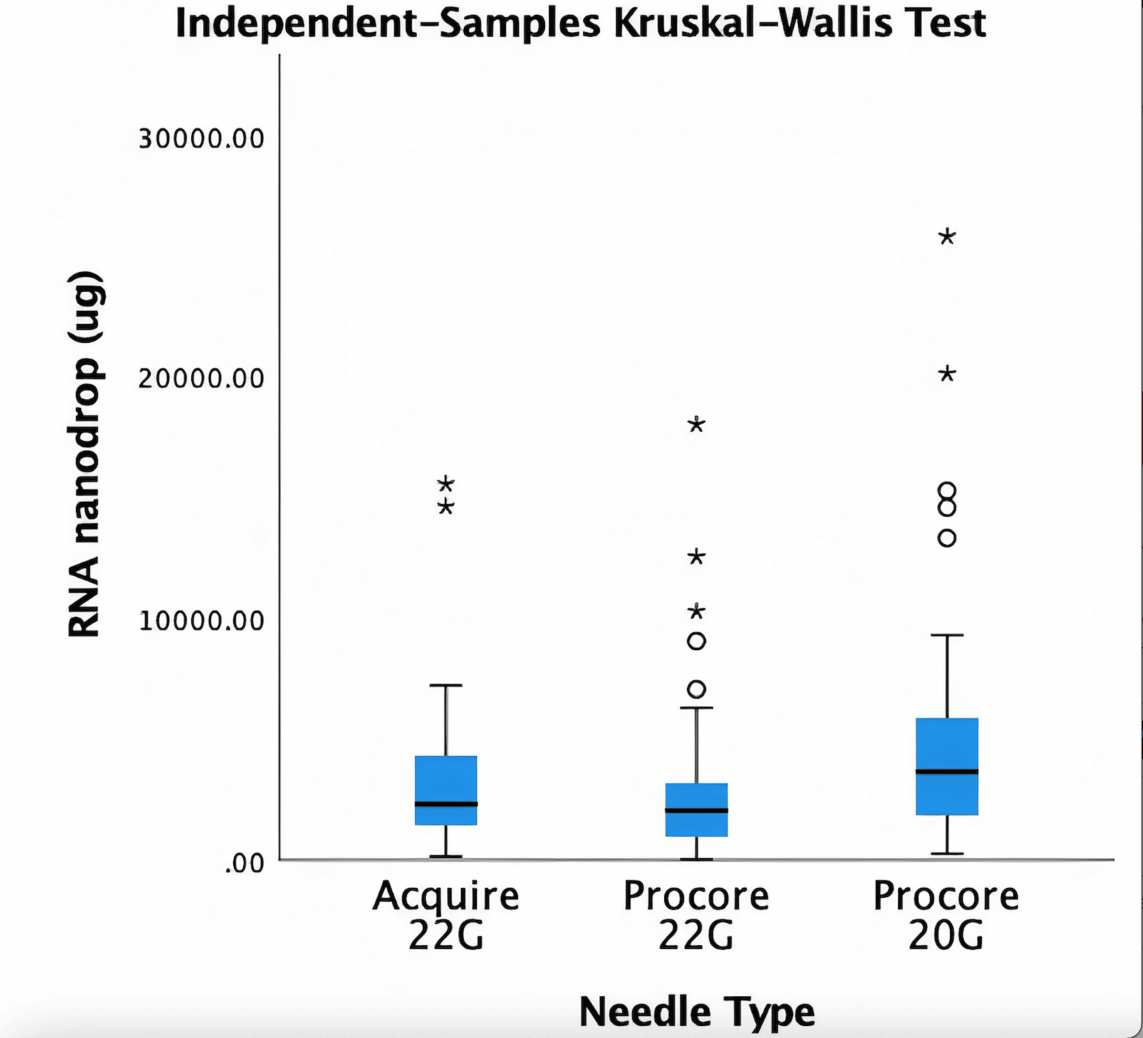
Kruskal Wallis test for RNA quantity RNA=Ribonucleic acid

**Figure 3:**
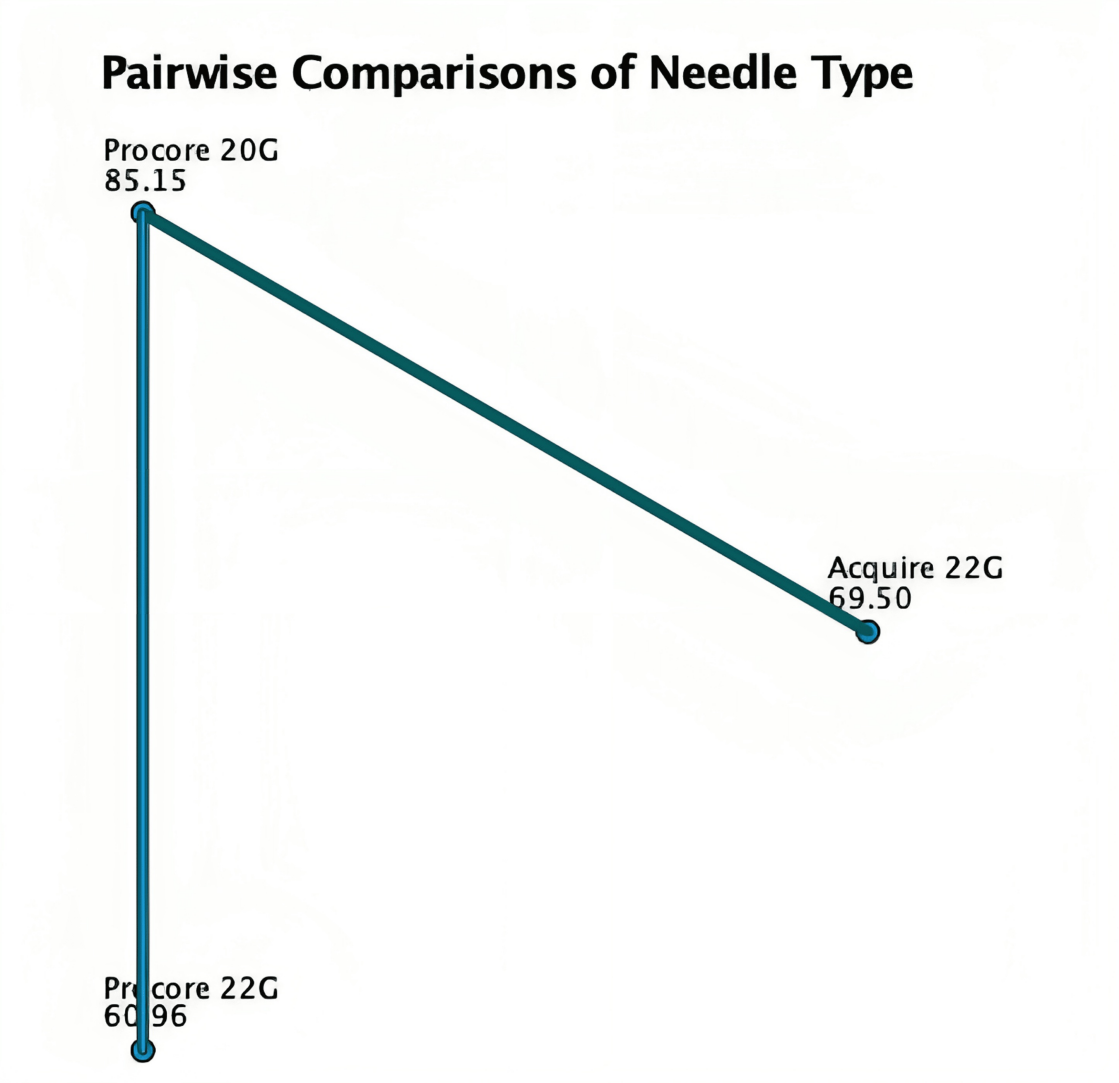
Pairwise comparison of NanoDrop against needles. Each node shows the sample average rank of needle type.

The median quantity from Acquire 22G was 2310.56µg (1439.25, 4312), ProCore 22G was 2050µg (966.4, 3181.6) and ProCore 20G was 4125µg (2003.75, 5954.75) (Table 3). Kruskal Wallis test demonstrated a statistically significant difference between needle sizes (Figure 2). Post hoc analysis utilising pairwise comparison demonstrated that ProCore 20G needle acquired more RNA than ProCore 22G (p = 0.004) and Acquire 22G (p = 0.065) (Figure 3).

**Table 3:** RNA quantity and quality results RNA=Ribonucleic acid; IQR=interquartile range

	Acquire 22G (n=43)	ProCore 22G (n=45)	ProCore 20G (n=57)	p-value
RNA Quantity (µg) (IQR)	2310.56 (1439.3, 4312)	2050 (966.4, 3181.6)	4125.8 (2003.8, 5954.8)	0.012
	Acquire 22G (n=25)	ProCore 22G (n=14)	ProCore 20G (n=45)	p-value
RNA Quality (RIN) (IQR)	2.95 (2.6, 4.9)	2.95 (2.5, 5.3)	2.7 (2.5, 3.5)	0.886

Of the 145 patients, RNA quality was available for 87 patients (A22G: 28, P22G: 14, P20G: 45). Acquire 22G had a median RIN of 2.95 (2.58, 4.9), ProCore 22G was 2.95 (2.5, 5.25), ProCore 20G was 2.7 (2.5, 3.5). Kruskal Wallis test did not identify any statistically significant difference (p = 0.520). The Pearson’s R correlation coefficient for RNA NanoDrop and RIN was 0.045 (p = 0.683) (Table 4).

**Table 4:** Post-hoc analysis Std. Error = standard error; Std. Test Statistic = standard test statistic

Sample 1-Sample 2	Test Statistic	Std. Error	Std. Test Statistic	p-value
ProCore 22G – Acquire 22G	8.544	8.957	0.954	0.34
ProCore 22G – Acquire 20G	-24.194	8.376	-2.889	0.004
Acquire 22G – ProCore 20G	-15.649	8.484	-1.845	0.065

Demographic and cytology findings for DNA

One hundred thirty-seven patients with a final diagnosis of PDAC were identified across three needle groups (Table 5) with DNA data: Acquire 22G (n = 39), ProCore 22G (n = 40) and ProCore 20G (n = 58). Median age between the groups ranged between 69 to 72 (p = 0.279). Acquire 22G had fewer females and ProCore 22G had fewer males (p = 0.062) (Table 5). 

**Table 5:** Demographic and cytology findings for patients with DNA data PDAC=Pancreatic ductal adenocarcinoma; DNA=Deoxyribonucleic acid; M=male; F=female

DNA	Acquire 22G (n = 39)	ProCore 22G (n = 40)	ProCore 20G (n = 58)	p-value
Median Age	69	73	72	0.279
Sex				
M, n (%)	24 (62)	14 (35)	28 (48)	0.062
F, n (%)	15 (38)	26 (65)	30 (52)	
Positive Cytology for PDAC, n (%)	34 (87)	34 (87)	48 (83)	0.837
Other Cytology findings				
Suspicious, n (%)	1 (3)	2 (5)	2 (3)	0.932
Atypical cells, n (%)	2 (5)	2 (5)	6 (10)	
Non-diagnostic, n (%)	2 (5)	2 (5)	2 (3)	

In total, 116 patients had positive cytology findings for PDAC, five with suspicious or highly suspicious features for PDAC, 10 with atypical cells without definite features of malignancy, and six with non-diagnostic cytology. Therefore, the sensitivity of EUS-guided biopsy in diagnosing solid pancreatic masses in this cohort was 84.7%. The distribution of positive cytology PDAC cases were similar among Acquire 22G (n = 34), ProCore 22G (n = 34) and ProCore 20G (n = 48) (p = 0.837). Non-PDAC cytology findings were similar between groups (p = 0.932).

DNA tumour characteristics

The majority of patients had either locally advanced (35.8%) or metastatic disease (45.2%) (Table 6). Over half (55.5%) of PDAC tumours were primarily located in the head. Other sites included neck (8.0%), body (15.3%), tail (7.3%), uncinate process (6.6%), distal pancreas (2.2%), and other (ampulla 1.5%). Onsite cytology was available for a significant higher proportion (95%) of ProCore 22G needle biopsies (p < 0.001).

**Table 6:** Tumour Characteristics DNA=Deoxyribonucleic acid; NCCN=National Comprehensive Cancer Network

DNA (n = 137)	Acquire 22G (n = 39)	ProCore 22G (n = 40)	ProCore 20G (n = 58)	p-value
NCCN Stage				
Operable, n (%)	0 (0)	6 (15)	4 (7)	0.326
Borderline resectable, n (%)	3 (8)	3 (3)	6 (10)	
Locally advanced, n (%)	15 (38)	15 (38)	19 (33)	
Metastatic, n (%)	19 (49)	16 (40)	27 (47)	
Tumour location				
Head, n (%)	17 (44)	25 (63)	34 (59)	0.279
Neck, n (%)	5 (13)	4 (10)	2 (3)	
Body, n (%)	8 (21)	3 (8)	10 (17)	
Tail, n (%)	3 (8)	2 (5)	5 (9)	
Uncinate, n (%)	5 (13)	2 (5)	2 (3)	
Distal, n (%)	0 (0)	1 (3)	2 (3)	
Other, n (%)	0 (0)	0 (0)	2 (3)	
Presence of onsite Cytology, n (%)	3 (8)	38 (95)	17 (29)	<0.001

DNA quality and quantity and correlation

The median quantity from Acquire 22G was 3340.5µg (1260, 6787.5), ProCore 22G was 2610.4µg (1899.5, 6062.1) and ProCore 20G was 3499.7µg (1445.8, 5947.3) (Table 7).

**Table 7:** DNA quantity and quality results DNA=Deoxyribonucleic acid;  IQR=interquartile range

	Acquire 22G (n = 43)	ProCore 22G (n = 45)	ProCore 20G (n = 57)	p-value
DNA Quantity (µg) (IQR)	3340.5 (1260, 6787.5)	2610.4 (1899.5, 6062.1)	3499.65 (1445.8, 5947.2)	0.763
	Acquire 22G (n = 22)	ProCore 22G (n = 22)	ProCore 20G (n = 31)	p-value
DNA Quality (DIN) (IQR)	7.3 (6.8, 7.5)	7.25 (6.7, 7.6)	7.5 (6.9, 7.6)	0.449

**Figure 4:**
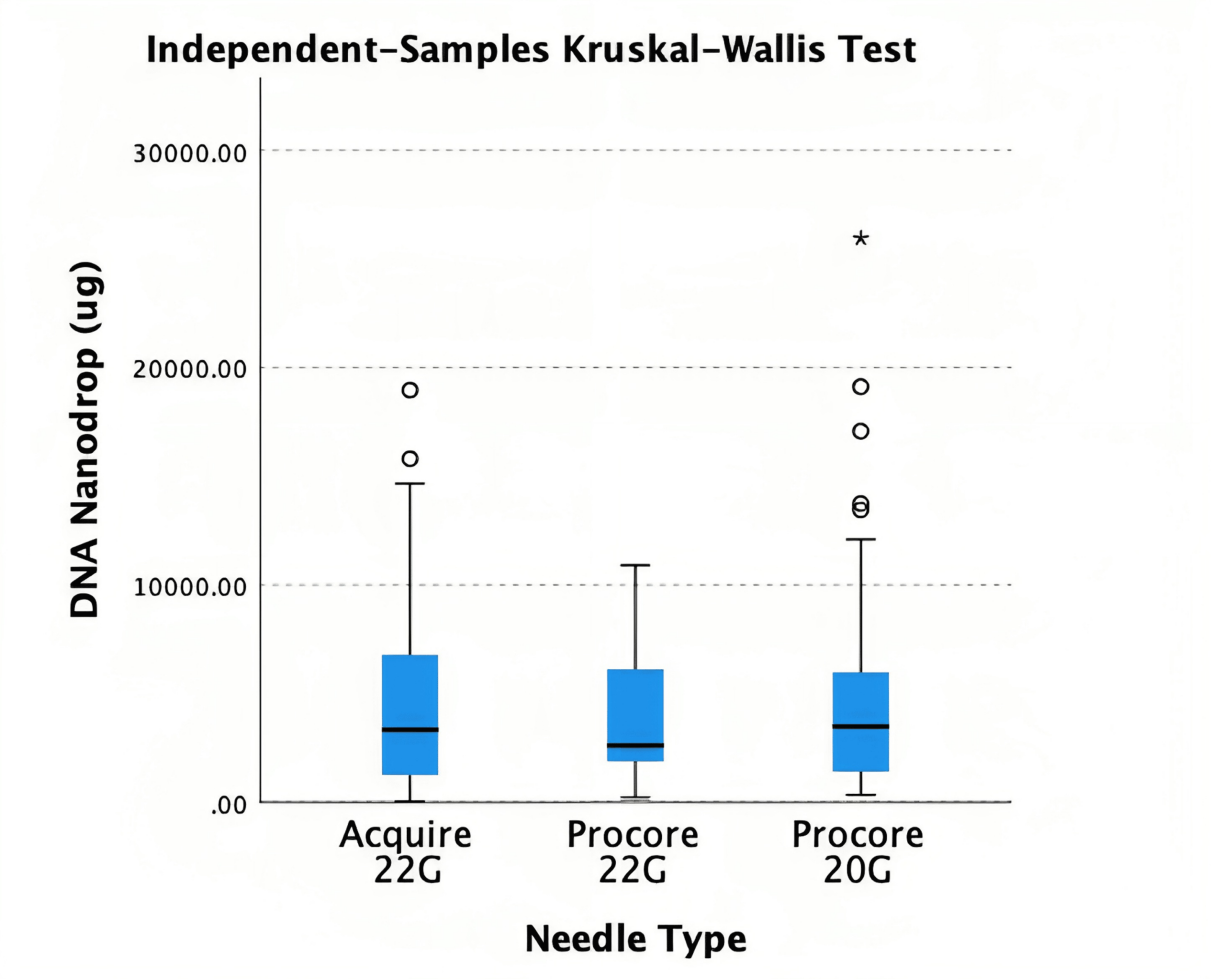
Kruskal Wallis test for DNA quantity DNA=Deoxyribonucleic acid

**Figure 5:**
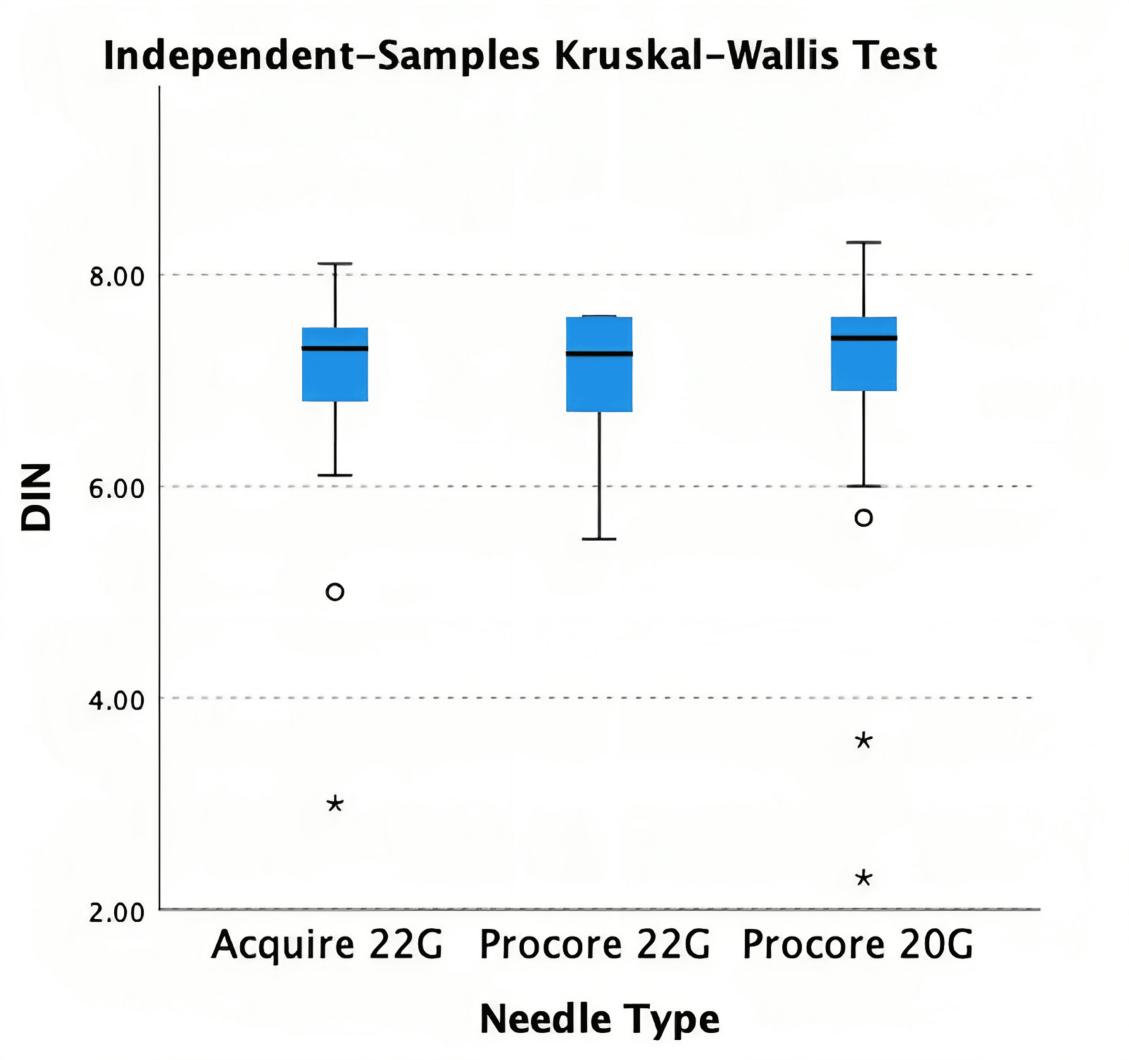
Kruskal Wallis test for DNA quality DNA=Deoxyribonucleic acid

Kruskal Wallis test demonstrated no statistically significant difference between needle sizes (p = 0.763) (Figure 4). Of the 137 patients, DNA quality was available for 75 patients (A22G: 23, P22G: 22, P20G: 30). Acquire 22G had a median DIN of 7.3 (6.8, 7.5), ProCore 22G was 7.3 (6.7, 7.6), ProCore 20G was 7.4 (6.7, 7.6). Kruskal Wallis test did not identify any statistically significant difference (p = 0.434) (Figure 5). Pearson’s R correlation coefficient for DNA NanoDrop and DIN was 0.089 (p = 0.449).

Correlation of needle extraction periods and RNA and DNA volumes over data period

**Figure 6:**
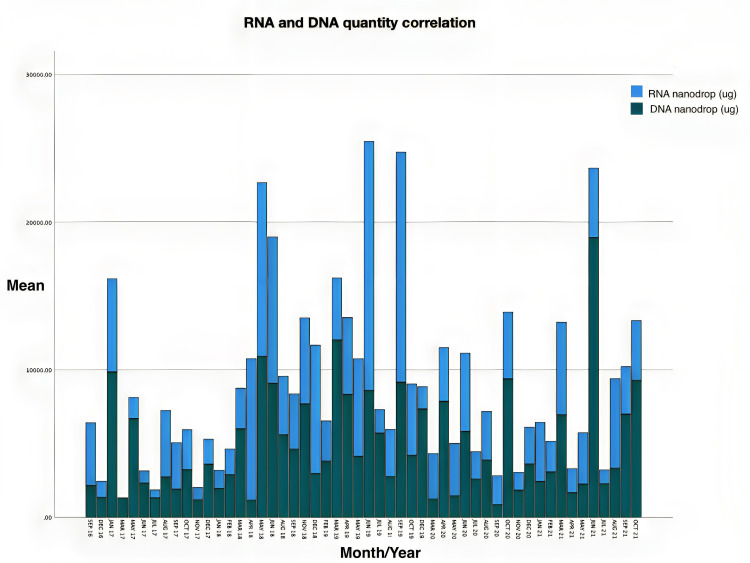
Graph showing RNA and DNA each belonging to the same patient over time. DNA=Deoxyribonucleic acid; RNA=Ribonucleic acid

To determine if endoscopic skills were associated with increased volume procured, data from patients who had both RNA and DNA collected were compared over time, from 2016 to 2021 (Figure 6).

**Figure 7:**
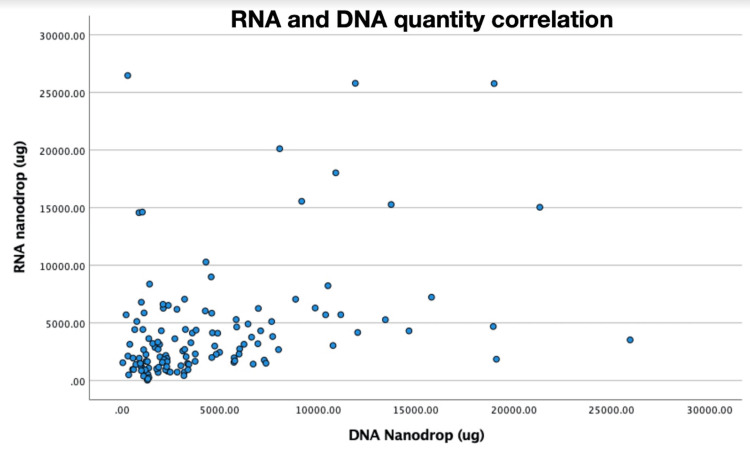
DNA and RNA correlation. DNA=Deoxyribonucleic acid; RNA=Ribonucleic acid

A scatterplot illustrating correlation of RNA to DNA in patients who had both taken is also shown (Figure 7). Pearson’s R correlation coefficient for RNA and DNA NanoDrop demonstrates a moderately positive correlation 0.383 (p < 0.001).

## Discussion

EUS-TA has been at the forefront of obtaining tissue diagnosis for PDAC over the past few decades. Tissue acquisition for precision medicine is becoming increasingly desirable for multiple reasons including diagnostication, prognostication and treatment direction. Especially now that guidelines recommend germline and somatic testing as standard management for PDAC, it further emphasises the need to clarify the procedural methodologies [27]. Being able to identify potential genetic biomarkers for targeted chemotherapy is beneficial for risk stratifying borderline resectable cases and down-staging disease, as well as surgically resectable PDAC for improving long-term outcomes.

The key findings in our study revealed that using a ProCore 20G needle during EUS biopsy was associated with a higher amount of genetic material compared with other needles (p = 0.0012). However, the Kruskal Wallis test does not discriminate to which needle the comparison is made. Post hoc analysis by pairwise comparison revealed that ProCore 20G collected more RNA tissue than ProCore 22G which was statistically significant (p = 0.001). Although it also procured more tissue than Acquire 22G, this was not statistically significant (p = 0.065). Comparison between Acquire 22G and ProCore 22G revealed no statistically significant difference (p = 0.340). In our cohort of patients, the sensitivity of EUS biopsies for PDAC was in keeping with the current literature at 83% [28] However, we separated suspicious (including highly suspicious) and atypical findings from a positive diagnosis as these are not significant to establish a confirmed diagnosis from a cytological or histological perspective alone [29]. Moreover, formal pathological diagnosis is increasingly recognised as an independent prognostic marker in PDAC [30]. In our series of 277 patients, five accredited endoscopists were involved. Four were trained and experienced endoscopists, while one was a fellow with less experience. The latter performed 15 procedures while the remaining were broken into 238, 14, seven and three respectively. No demonstrable pattern of increase or decrease in tissue acquisition was seen over the five-year period at any time point suggesting that even though different needles were used during different time periods, technical skill unlikely played a significant role. 

We expected that the volume of tissue would be proportional for both DNA and RNA and found that there was a moderate correlation (Pearson’s R = 0.383, p < 0.001). On further analysis, it was found that a higher proportion of samples had higher DNA yet correspondingly lower RNA (Figure 7), with the exception of the ProCore 20G needle. Indeed, median DNA compared to RNA was higher for both Acquire 22G (3340µg vs 2310µg) and ProCore 22G (2610µg vs 2050µg). There are two reasons that may explain this. Firstly, both DNA and RNA are chemically different. RNA is more chemically labile and therefore, it breaks down quicker and is gathered in lower quantity and quality. Secondly, DNA is more likely to be collected in whole blood due to the presence of ribonuclease (RNase) in leukocytes and the pancreatic gland, which is abundant in RNase. Therefore, in the procurement process, quality may be detrimentally affected [26]. 

Fine needle aspiration (FNA) vs fine needle biopsy (FNB)

FNA, which uses a traditional suction to draw up cellular material for cytological analysis, had previously been the mainstay of EUS sampling [31]. FNB needles differ in that they perform a core biopsy of the tissue, thereby maintaining cellular architecture and stromal integrity and allowing for histological analysis [32,33]. On a broader level, FNA is not a comprehensive diagnostic tool, and struggles to differentiate other conditions such as autoimmune pancreatitis, lymphoma and well-differentiated adenocarcinoma. It can only be evaluated on a cytological level, and the ability to perform ancillary testing (immunohistochemistry, molecular tumour profiling) is limited [34,35]. For this reason, and owing to the small amount of FNA needles in our study cohort (n=18) of various gauges (19G, 20G, 22G and 25G) we excluded it from analysis.

In terms of tissue acquisition for theranostic purposes, few retrospective studies have been published. Most articles lacked quantification and qualification of genetic tissue [17,36,37]. Karsenti and colleagues measured cored tissue length and surface area as a surrogate for histological quantity comparing 22G Acquire to 20G ProCore. Although they found that Acquire 22G procured more physical tissue than ProCore 20G, no formal genetic analyses were conducted [38]. Yoshizawa et al. compared 22G against 25G needles and found that both were suitable for immunohistochemistry analysis as a surrogate for molecular profiling. However, their study did not separate FNA from FNB needles, which differ fundamentally as aforementioned [36]. Elhanafi et al. demonstrated that FNB was superior to FNA in acquiring sufficient samples for genomic analysis (90.9% vs 66.9%; p = 0.02). On further analysis, their multivariable model showed that FNB was (OR 4.95, 95% CI 1.11 - 22.05, P = 0.04) the only factor associated with sufficient sampling. We note that their modelling investigated a binary outcome of sufficiency rather than overall volume. Although it could also be argued that in translation to clinical practice, sampling sufficiency is more clinically relevant. However, their study was small. Of the 167 patients in their study, 145 underwent FNA, and only 22 patients had FNB samples. Needle gauge and design were also not assessed as all cases used a 22G needle of various brands [17]. 

Few studies have been published in regard to quantifying genomic yield and quality [18,26,39]. In their randomised crossover trial of 50 patients, Kandel et al. investigated DNA concentrations in 50 PDAC patients and found that FNB procured more genetic material than FNA (5.930 µg/mL vs 3.365 µg/mL, p = 0.01). However, FNA needles were smaller (25G), and FNB needles (19G or 22G) were not stratified according to design or size. Furthermore, only DNA was investigated in their study [39]. In another study by Archibugi et al. looking at RNA quality and quantity, the team concluded that neither FNA nor FNB made any difference to their endpoint. Again, their study was plagued by multiple important factors including small sample size (n = 37), high variability in needle size and design (25G FNA, 20G and 25G FNB), as well as different fixation methods (Trizol, snap frozen and RNALater) [26]. Nonetheless, these pilot studies provided a promising start to the utility of FNB in advancing precision medicine.

Needle size and design

**Figure 8:**
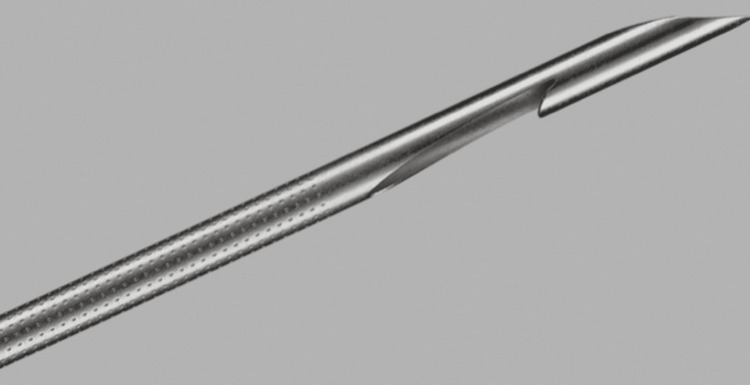
ProCore reverse bevel Westcott; Cook Medical

**Figure 9:**
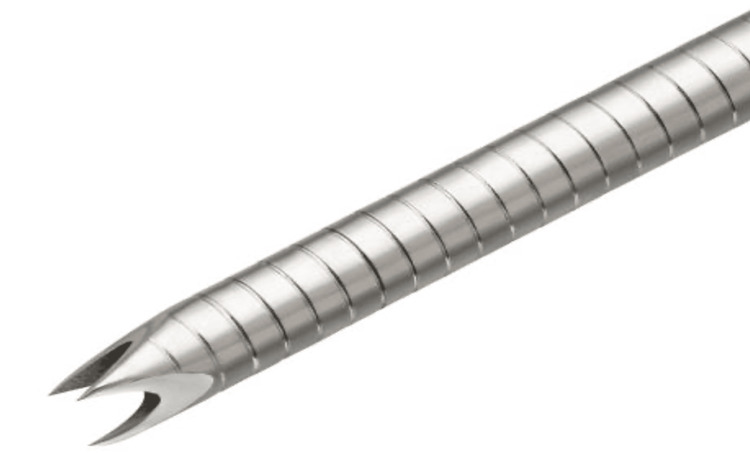
Acquire three-pronged “Franseen” style needle; Boston Scientific

The last decade has seen a significant paradigm shift to FNB owing to its superiority in diagnostic accuracy as well as enabling histological analysis [40]. ProCore introduced a flexible, reverse bevel Westcott design, in 19G, 22G and 25G (Figure 8) allowing for easier manoeuvrability during transduodenal biopsies. The three symmetrical plane, or Franseen, style needle seen in the Acquire 22G (Figure 9) utilised cutting edges to enter tissue and the cutting tips to remove the core biopsy. Studies showed that these two performed well and yielded better histological and cytological value than FNA, with fewer passes [41,42]. In addition, Bang et al. showed that when compared to FNA, there were no significant differences between diagnostic yield (96% vs 92%, p = 0.32) and diagnostic adequacy with rapid onsite evaluation (ROSE) (94% vs 96%, p = 0.32), thus potentially obviating the need for ROSE [43]. 

**Figure 10:**
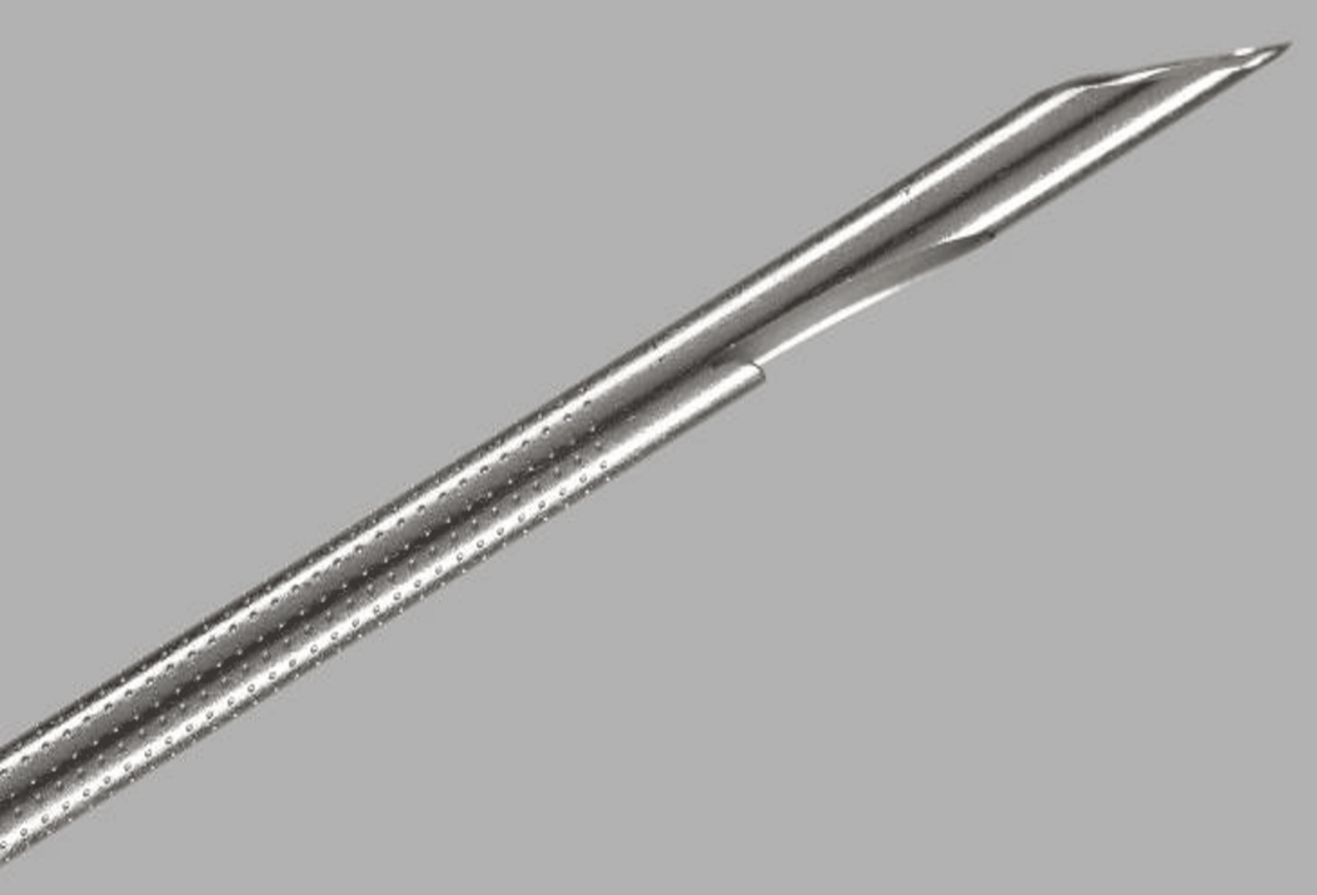
ProCore 20G forward bevel; Cook Medical

The ProCore 20G was another different design that differed slightly from its 19G, 22G and 25G counterparts. It recruits a forward bevel Westcott design (Figure 10). A retrospective study of 191 patients by Watanabe et al. showed the ProCore 20G had superior diagnostic accuracy compared to ProCore 22G (96.4% vs 72.1% p<0.0001) but was comparable to the Acquire 22G [44]. Larger gauge is the first reason. Controlling for other technical factors, larger gauge needle procures more tissue and also protects the histological integrity of the core biopsy due to a thicker, non-exposed core (2.9mm vs 2mm) while maintaining similar stroke lengths (3.8mm vs 3.9mm) and hence geometrical volumes - which would reasonably aid diagnostic accuracy [34]. The second reason may be attributed to the forward bevel core trap design of the 20G ProCore. This novel characteristic is due to the presence of a side bevel with an antegrade cutting edge, enabling procurement of a higher tissue volume as it catches the tissue on advancement compared with the reverse bevelled needles [32]. Armellini et al. confirm this idea that the design allows better tissue catchment while the needle is advanced. In their research combining 238 patients the 20G ProCore procured more histologic-grade tissues than the 22G ProCore (92.6% vs 49.5%, p < 0.0001). They also achieved this using a lower number of passes (2.64 vs 3.44, p < 0.0001) [45].

Larger needle calibre is known to correlate positively with increasing histologic value, especially in studies involving 19G ProCore needles [46]. Despite this, its use has declined over time, suggesting a limitation of using a wide-bore needle. Namely, higher complication rates and difficulty in manipulation. Due to the larger gauge, manoeuvring a needle around a corner during EUS is difficult. This increased difficulty leads to increased risks, poorer performance and targeting of the pancreatic lesion, and subsequent indeterminate results. This is due in part to technical difficulty as well as poor manoeuvrability resulting in poor sensitivity (70.7%) and diagnostic accuracy (73.6%). The main reason for technical failure was the inability to place the scope in the proper position. Major complications occurred in six (2.5%) cases, significantly more than EUS FNB [47]. Given most studies and centres primarily employ 20G or 22G needles, practice patterns would suggest there is a balance between form and function.

Regarding gauge and histological analysis, only one study has been identified in the literature [18]. Park and colleagues identified 109 patients in whom next-generation sequencing (NGS) was able to be completed. They found that larger needle gauge (19G or 22G) was the only factor associated with successful NGS. Their study utilised the ProCore reverse bevel Westcott needle in 19G, 22G and 25G, as well as FNA 19G, 22G and 25G needles. As such, this introduced a large variability, effectively introducing six different types of needles. No subset analysis was done between 19G and 22G of different styles, presumably due to low patient numbers [18]. 

Current status of precision medicine in PDAC

Few studies have looked at tailoring chemotherapeutics to genetic profiles. This is because of the swift progression of PDAC, difficulty attaining timely molecular profiling, and ethical and logistical delays in enrolling patients. Notably, the phase two IMPaCT (Individualised Molecular Pancreatic Cancer Therapy) trial, failed to formally enrol any patients to their study. Among their many hurdles was the difficulty in attaining high-quality tissue in adequate yields to enable molecular profiling [16]. The COMPASS (Comprehensive Molecular Characterisation of Advanced Pancreatic Ductal Adenocarcinoma for Better Treatment Selection) trial was able to perform prospective whole genomic sequencing on 63 patients. However, their study utilised radiologically guided percutaneous core biopsies on all patients rather than EUS FNB. Radiologically guided percutaneous core biopsy for pancreatic lesions is more technically difficult. Lesions under 10mm are often not amenable to percutaneous core biopsies, and complication rates are markedly higher, up to 8.6% compared to 1% in EUS-FNB [48]. This fundamentally excludes a large proportion of patients - not dissimilar to the initial issue of analysing ex vivo surgical specimens only [49]. 

The above studies favour the notion that molecular profiles may be associated with better outcomes using particular treatments, even though they were not matched per se. The largest retrospective study to date using a nationwide registry Know Your Tumour, explored precision medicine for PDAC in much greater detail. Pishvaian et al. enrolled 320 patients who had actionable genomic alterations. Those who received matched therapy had a significantly longer median progression-free survival (PFS) than those who received unmatched therapy [50]. In their subsequent study investigating overall survival, they recruited 189 patients with actionable profiles - 46 (24%) received target-matched treatment and 143 (76%) received unmatched treatment. They successfully demonstrated that overall survival was significantly longer in the matched therapy group (2.58 years vs 1·51 years, p=0·0004) [51]. 

To date, the first prospective biomarker selected study conducted by Lundy and colleagues demonstrated a potential role of EUS biopsies and molecular profiling and translating it to clinical practice in their feasibility trial. Eight patients with KRAS wild-type PDAC were selectively treated with panitumumab. 14.3% had four months PFS - although no objective tumour responses were observed [52]. Given the likelihood that many patients harbour multiple genetic pathways for persistent tumour signal activation, finding the main molecular aberrations and treating with multiple therapeutic agents would be crucial in future studies [53]. Multiple barriers must be overcome in order to obtain high-quality molecular testing for patients with PDAC, then identify potential actionable alterations and then get patients access to a suitable therapy. Adding to this complexity is the timing of tissue acquisition and molecular profiling which can take up to two months; noting that median PFS in multiple cohorts of unmatched therapy groups is less than two months. These studies add to the body of research confirming the diagnostic and stratifying utility of EUS biopsy as a reliable source of tumour material for molecular analysis.

Limitations

Our study had several limitations. Firstly, this is a single-centre retrospective cohort study that would be inherently subject to biases. Because our study collected four discrete data variables (quality and quantity of DNA and RNA), data was missing for a significant proportion of the patients, especially for qualitative data. Second, although we found that ProCore 20G procured more RNA material than ProCore 22G and Acquire 22G, all three needles were different in both size and style. Perhaps larger gauge, the forward bevel Westcott style, or both were the associative factors attributed to our finding. Lastly, the number of passes was a factor that was not analysed in our study. The decision to use only a single pass means our results may not fully represent scenarios where multiple passes are performed. In clinical practice, multiple passes are often used to increase the likelihood of obtaining adequate material, particularly for challenging lesions. Therefore, while our findings are relevant for single-pass procedures, they may not be directly applicable to situations where multiple passes are routinely performed. The potential effects of multiple passes on tissue yield, sample quality, and patient safety remain unexplored in our study and warrant further investigation.

## Conclusions

Our study demonstrated that ProCore 20G was associated with acquiring a higher quantity of RNA tissue than ProCore 22G (p = 0.004) and Acquire 22G although the latter was not statistically significant (p = 0.065). Overall sensitivity of EUS FNB in our cohort was in line with the literature at 83%. This is the first study to date investigating the impact of biopsy needles on both quality and quantity of DNA and RNA. Future research should explore the impact of the number of needle passes on genetic material yield and quality. There is a need to compare different numbers of passes across various needle gauges to find the optimal balance between obtaining sufficient genetic material and minimizing procedural risks. Additionally, standardizing needle types and biopsy techniques could reduce variability and improve the consistency of results. Developing guidelines for selecting needle size based on specific factors, such as lesion type and patient characteristics to achieve higher genetic quality and quantity in EUS-TA for advancing precision medicine.
